# Double pituitary adenomas in a large surgical series

**DOI:** 10.1007/s11102-019-00996-2

**Published:** 2019-10-09

**Authors:** Grzegorz Zieliński, Emir Ahmed Sajjad, Maria Maksymowicz, Monika Pękul, Andrzej Koziarski

**Affiliations:** 1 Department of Neurosurgery, Military Institute of Medicine, Warsaw, Poland; 2 Department of Pathology and Laboratory Diagnostics, M. Skłodowska-Curie Memorial Cancer Centre and Institute of Oncology, Warsaw, Poland

**Keywords:** Double pituitary adenoma, Surgical treatment, Transsphenoidal approach, Immunohistochemistry

## Abstract

**Purpose:**

To explore the incidence of double pituitary adenomas in a tertiary center for pituitary surgery and asses their clinical, imaging and histopathological features.

**Methods:**

The medical records of the patients operated on for pituitary tumors at the Department of Neurosurgery of Military Institute of Medicine in Warsaw, Poland between the years 2003 and 2018 were retrospectively analyzed. Among the 3270 treated patients, the diagnosis of double pituitary adenoma was established in 22 patients. Clinical, laboratory, detailed histopathological and diagnostics imaging data were collected and analyzed.

**Results:**

There were 21 cases of synchronous and one case of asynchronous double pituitary adenoma. The main clinical finding was acromegaly (12/22) followed by Cushing’s disease (3/22). The diagnosis of synchronous double pituitary adenoma was suspected in the preoperative MRI in 11 patients. In the remaining patients the diagnosis of contiguous double pituitary adenoma was confirmed in the histopathological examination. There was no predilection for gender and the mean observation time was 74.2 months. In one case of Cushing’s disease the occurrence of double pituitary adenoma led to the initial failure of achieving hormonal remission. One patient presented with double pituitary adenomas as a manifestation of Carney complex.

**Conclusions:**

Double pituitary adenoma is a rare entity that can pose a significant challenge especially in the setting of Cushing’s disease. Careful inspection of preoperative MRI and diagnostic work-up before transsphenoidal surgery and thorough histopathological microscopic examinations with immunohistochemical staining for all pituitary hormones is essential for establishing the diagnosis of double pituitary adenoma.

**Electronic supplementary material:**

The online version of this article (10.1007/s11102-019-00996-2) contains supplementary material, which is available to authorized users.

## Introduction

Pituitary adenomas are mostly benign tumors arising from the anterior pituitary gland. They are the third most common intracranial neoplasms after gliomas and meningiomas, accounting for approximately 15% of all intracranial tumors and have been identified with a population prevalence of ~ 80/100,000 [1–3]. The majority of these tumors are monoclonal and correspond to a specific pituitary cell type. However, up to one third of pituitary adenomas express more than one pituitary hormone and are therefore designated as plurihormonal [4]. Plurihormonal adenomas are further divided into monomorphous adenomas consisting of one cell type that is capable of producing two or more hormones and plurimorphous adenomas composed of two or more cell types, each producing different hormone [4]. Rarely, synchronous true double or multiple pituitary adenomas are encountered. Double and multiple pituitary adenomas according to their morphological appearance are divided into clearly distinct tumors, recognized as such by neuroradiological imaging and/or intraoperatively and contiguous tumors, which are resected as a single tumor and confirmed as multiple pituitary adenomas in the histopathological examination [5]. The occurrence of multiple pituitary adenoma increases the risk of surgery failure in treating anterior pituitary gland syndromes, as the hormonally active lesion may be left behind during surgical exploration [6, 7]. Rarely, multiple pituitary adenomas appear not synchronically but as a recurrence of previously resected pituitary adenoma with distinct immunohistochemical profile [8]. The aim of this study is to report a case series of double pituitary adenomas in a high volume tertiary pituitary surgical center with special references to diagnostic challenges, treatment pitfalls and endocrinological results.

## Materials and methods

A retrospective review of patients operated on for pituitary adenoma from 2003 to 2018 at the Neurosurgery Department of Military Institute of Medicine in Warsaw was performed. Patients’ complete medical and imaging records were analyzed. All patients underwent full pre- and postoperative hormonal and ophthalmological evaluation and magnetic resonance imaging. MR imaging protocol included a T2-weighted 1.5 T scanning in coronal sections and T1-weighted contrast enhanced and non-enhanced 1.5 T scanning in coronal, sagittal and axial sections. In the setting of Cushing’s disease, 3 T SE and SPGR MR imaging with contrast enhancement were performed whenever the first, 1.5 MRI imaging, was non-conclusive.

Preoperative endocrine assessment consisted of serum PRL, TSH and free thyroid hormone levels (thyroid axis), IGF-1 and GH (growth hormone axis), LH, FSH and estradiol or testosterone (gonadotropin axis) and random morning cortisol and ACTH (adrenal axis) measurements. Additionally following dynamic tests were performed: cortisol circadian rhythm, 1 mg ODST (overnight dexamethasone suppression test, 1 mg at midnight), 24-UFC (24 h urinary free cortisol), HDDST (high dose dexamethasone suppression test; 2 mg q.i.d for 48 h), BIPSS (bilateral inferior petrosal sinus sampling, 100 µg CRH) for Cushing’s disease, GH in oral glucose tolerance test (for acromegaly) and TRH stimulation test (for secondary hyperthyroidism).

Ophthalmological evaluation including visual acuity test and automatic visual field test was performed pre- and postoperatively.

### Imaging characteristics

Pituitary tumors were classified as microadenomas (diameter ≤ 10 mm) or macroadenomas (at least one diameter > 10 mm). Based on preoperative MR images, the cavernous sinus invasion was graded according the Knosp scale [9]. Tumors with Knosp grades 0 and 1 were unlikely to enfold the cavernous sinus and have a favorable prognosis. In contrast, higher Knosp graded (2–4) usually indicate cavernous sinus involvement and a risk of relapse after surgery.

All patients underwent microscopic transsphenoidal resection of pituitary adenomas performed by one neurosurgeon.

### Pathological evaluation

Removed tissue was fixed in 10% formalin, embedded in paraffin, and routinely stained with hematoxylin and eosin (H&E). Immunohistochemical (IHC) staining was performed on paraffin embedded specimen according to the labeled EnVision Flex Visualization System (Dako, K8000) with 3,3′-diaminobenzidine (DAB) as chromogen using antibodies against anterior pituitary hormones or subunits: PRL (dilution 1:200, Neo Markers), GH (dilution 1:500, Neo Markers); ACTH (dilution 1:500, ThermoFisher), β-TSH (dilution 1:200, ThermoFisher), β-FSH (dilution 1:500, ThermoFisher), β-LH (dilution 1:200, ThermoFisher); the glycoprotein α-subunit (dilution 1:1000, Bio-Rad); somatostatin receptors: sstr2A and sstr5 (dilution 1:1500, BioTrend); p53 and Ki-67 (MIB1 clone, RTU, Dako). MIB-1 labeling index was established (<3% or ≥ 3% of positive nuclei). For hormone negative adenomas, in order to establish the diagnosis of null cell adenoma, transcription factors immunostaining with the following antibodies was performed: PIT-1 (dilution 1:100, ThermoFisher) and TBX19 (dilution 1:50, ThermoFisher) and steroidogenic factor 1 (dilution 1:200, Abcam).

For electron microscopy, small pieces of tissue were fixed in 2.5% glutaraldehyde, postfixed in 1% osmium tetroxide, dehydrated and embedded in Epon. Ultrathin sections were counterstained with uranyl acetate and lead citrate and examined with a Philips CM120 BioTWIN electron microscope.

### Postoperative hormonal assessment and criteria for remission

All patients were evaluated for anterior pituitary insufficiency and diabetes insipidus.

In Cushing’s disease; an immediate, postoperative remission was defined as a nadir morning serum cortisol level taken at 6 a.m. on the first, second or third postoperative day lower or equal to 1.8 µg/dL. Early biochemical remission was recognized as a clinical and biochemical evidence of adrenal insufficiency or in case of preserved adrenal function, biochemical evidence of eucortisolemia: UFC, morning serum cortisol and plasma ACTH levels within reference ranges, preserved circadian rhythm of serum cortisol and ODST-induced serum cortisol suppression to ≤ 1.8 µu/dL,

In PRL-secreting adenomas, a lack of hyperprolactinemia sings and symptoms and normalization of serum prolactin concentration (< 23 ng/mL in women and < 16 ng/mL in men), in GH- secreting adenomas, a random serum GH level < 1.0 µg/L, serum IGF-1 level in the sex- and age-adjusted normal range and nadir serum GH levels less than 1.0 µg/L in OGTT performed at least 12 weeks after surgery and in TSH-secreting adenomas, a decrease in serum TSH level below 0.1 mIU/L on the first, second or third postoperative day with decrease in serum fT4 and fT3 levels and postoperative TRH stimulation test performed 6 months after surgery were considered respectively as remission criteria that suggested completeness of tumor resection.

## Cases

A diagnosis of double pituitary adenomas was established in 22 patients from between 3270 cases of pituitary tumors operated on between the years 2003 and 2018 at our neurosurgical unit by one neurosurgeon. The diagnosed tumors were divided into four categories presented in Table 1. Case profiles are summarized in Table 2.

**Table 1:** Types of double pituitary adenomas

Symbol	Description
A	Clearly separate tumors identified in the preoperative MRI and intraoperatively with distinct histopathological diagnosis
B	Clearly separate tumors identified in the preoperative MRI and intraoperatively with similar histopathological diagnosis
C	Tumors clearly separated in histopathological examination but without distinguishable distinct masses in the preoperative MRI or intraoperatively (contiguous)
D	Two tumors with different histopathological features in the same patient but occurring over a time interval

**Table 2:** The overview of patients operated on with an established diagnosis of double pituitary adenomas

Case no.	Age, Gender	Tumor type^a^	Clinical symptoms
1	40, M	A	Acromegaly
2	60, F	A	Acromegaly
3	26, F	A	Hiperprolactinemia, secondary amenorrhea
4	47, M	A	Acromegaly, subclinical ACTH-dependent hypercortisolemia
5	66, M	A	Acromegaly
6	65, F	C	Cushing’s disease
7	13, F	C	Cushing’s disease
8	59, M	A	NFPA, bitemporal hemianopsia
9	41, M	A	Acromegaly
10	46, M	C	Acromegaly
11	70, F	C	Acromegaly
12	28/31^b^, M	D	Acromegaly/NFPA
13	62, M	B	NFPA
14	65, M	C	Secondary hyperthyroidism
15	36, F	C	Acromegaly
16	66, F	B	NFPA, bitemporal hemianopsia
17	63, F	C	Acromegaly
18	63, F	A	Acromegaly
19	45, F	A	Cushing’s disease
21	42, F	C	NFPA, bitemporal hemianopsia
20	19, M	A	Acromegaly, Carney complex (confirmed by clinical picture and genetic investigation)
22	53, F	B	NFPA

^a^See Table 1

^b^The first time the patient was operated on at the age of 28 due to GH-secreting adenoma causing acromegaly and then at the age of 31 because of another pituitary tumor recognized as a NFPA

The main clinical finding was acromegaly (12/22) followed by Cushing disease (3/22). There was one case of secondary hyperthyroidism and one case of hyperprolactinemia with secondary amenorrhea. Three patients had clinically non-functioning pituitary adenoma (NFPA) that was diagnosed due to bitemporal hemianopsia. One patient (case 12) at the age of 28 was operated on for an adenoma presenting with acromegaly. At the age of 31 in his control MR study a diagnosis of adenoma regrowth was suspected, however he did not meet endocrinological criteria of recurrent acromegaly. The excised specimen was FSH, LH and α-subunit immunopositive.

**Fig. 1:**
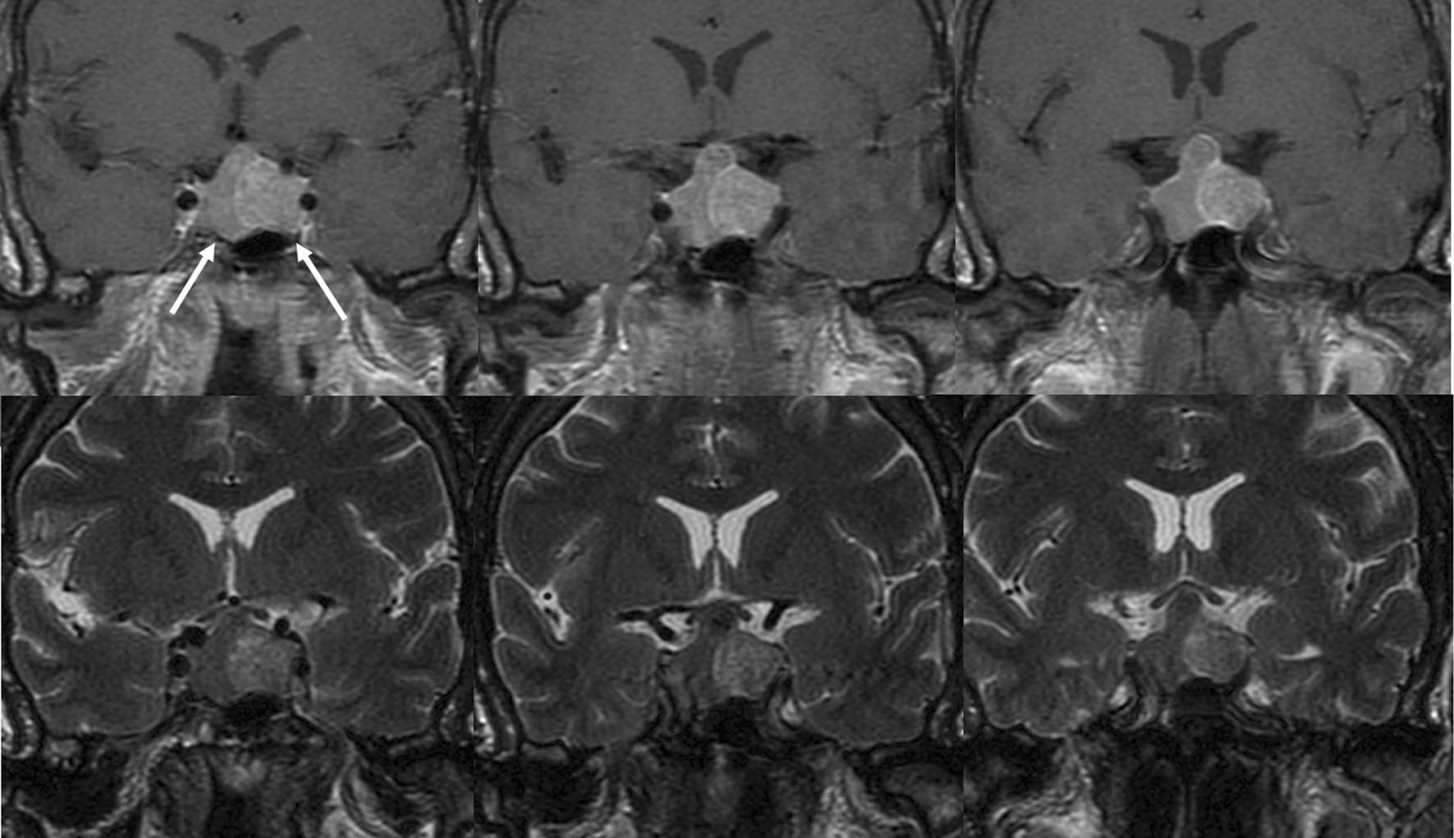
Case no. 1, coronal T1 (upper row) and coronal T2 (lower row) weighted MR imaging identified two clearly separate pituitary macroadenomas with different signal intensity. Left and right arrows indicate respectively somatotroph and gonadotroph tumors. Both tumors were separated by flattened pituitary gland (visible on T1 weighted MR imaging as a hyperintensive band between the two tumors and confirmed during transsphenoidal operation)

**Fig. 2:**
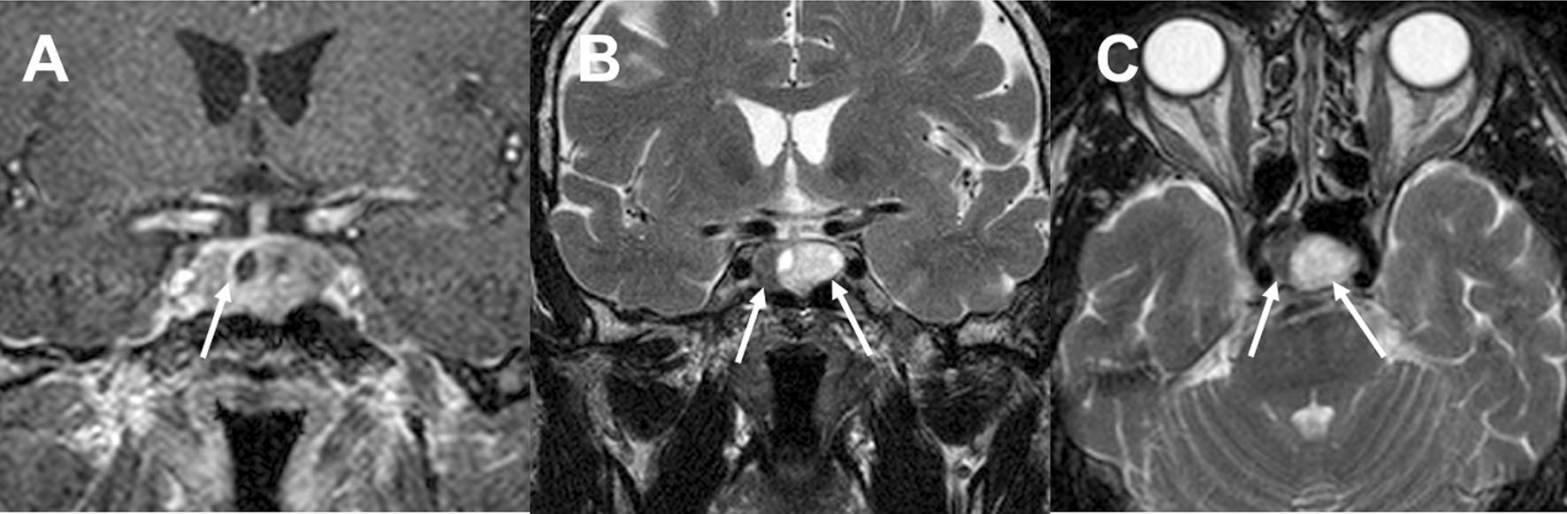
Case no. 2: **a** coronal T1 weighted MRI after Gd-DTPA demonstrates two clearly separate pituitary adenomas with flattened normal pituitary gland (arrow), **b** and **c** coronal and axial T2 MRI show gonadotroph macroadenoma with cystic degeneration (on the left side) and solid somatotroph adenoma (on the right side)

**Fig. 3:**
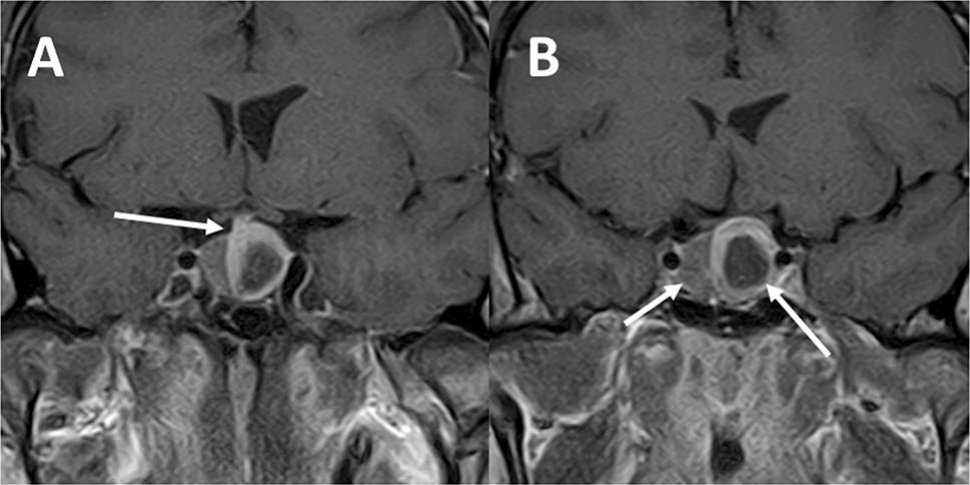
Case no. 9: **a** coronal T1 weighted MRI after Gd-DTPA revealed two clearly separate pituitary adenomas, **b** left arrow indicates intrasellar gonadotroph pituitary macroadenoma and right arrow shows cystic somatotroph macroadenoma with suprasellar extension

The diagnosis of synchronous double pituitary adenomas was suspected in the preoperative MRI in 11 patients and intraoperatively two separate tumors were identified in all of them (Figs. 1,2 and 3). In case 19 no tumors were visible in the preoperative MRI, however surgical exploration revealed two tumors with distinct histopathological features. The preoperative MRI conclusions and intraoperative findings are summarized in Table 3.

**Table 3:** Preoperative MRI and intraoperative findings

Case no.	MRI Findings					
	Visible separate tumors	Dimensions (mm)		Knosp scale		Intraoperatively distinguishable tumors
		Left	Right	Left	Right	
1	Yes	18 × 20 × 20	13 × 20 × 29	2	2	Yes
2	Yes	5 × 6 × 6	9 × 10 × 18	1	3	Yes
3	Yes	7 × 7 × 7	5 × 6 × 10	0	1	Yes
4	Yes	4 × 8 × 9	3 × 3 × 3	0	0	Yes
5	Yes	14 × 15 × 20	6 × 10 × 11	2	2	Yes
6	No	5 × 5 × 5		0		No
7	No	20 × 20 × 17		2		No
8	No	47 × 30 × 35		2		No
9	Yes	18 × 23 × 24	9 × 18 × 20	1	1	Yes
10	No	11 × 12 × 14		1		No
11	No	8 × 12 × 11		0		No
12	Yes^1^	16 × 10 × 13		2		
13	Yes	13 × 14 × 19	11 × 14 × 19	2	1	Yes
14	No	10 × 7 × 7		0		No
15	No	4 × 5 × 9		0		No
16	Yes	13 × 10 × 14	16 × 20 × 25	3	4	Yes
17	No	6 × 8 × 11		1		No
18	Yes	4 × 6 × 9	10 × 10 × 16	1	2	No
19	No^2^					Yes
20	No	42 × 35 × 27		2		No
21	Yes	4 × 4 × 4	3 × 3 × 3	0	0	Yes
22	Yes	18 × 11 × 9.2	17 × 12 × 9.5	1	2	Yes

The only case of asynchronous double pituitary adenomas. No tumors were not visible in the preoperative MRI, however surgical exploration revealed two tumors with distinct histopathological findings

**Fig. 4:**
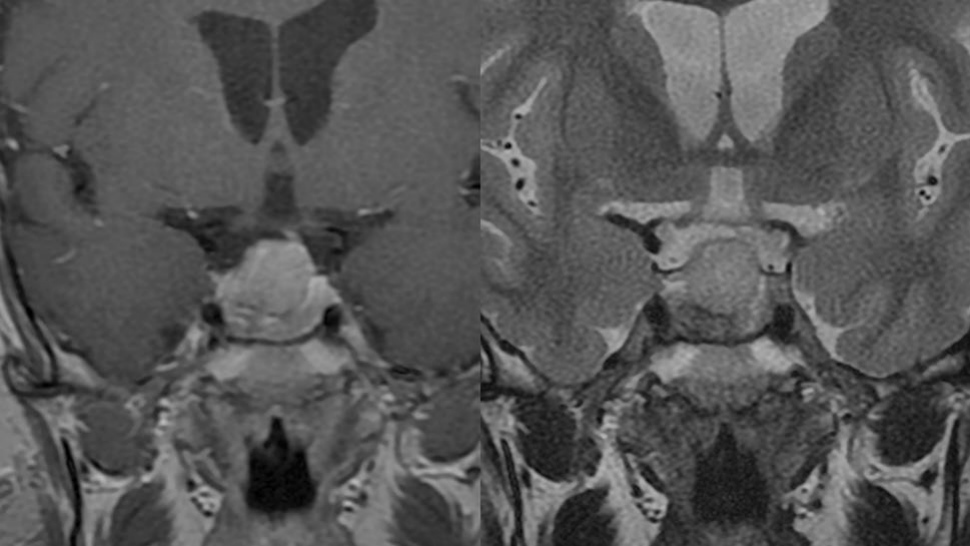
Case no. 7, coronal T1 weighted after Gd-DTPA (left image) and T2 weighted (right image) MR imaging revealed invasive pituitary macroadenoma invading sellar floor

**Fig. 5:**
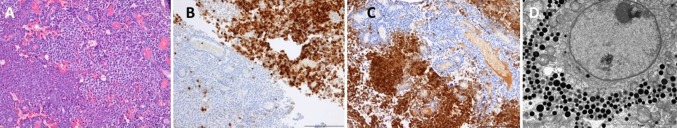
Contiguous double pituitary adenoma: case no. 7, Cushing disease. Pathomorphological evaluation: **a** H&E, **b** IHC for GH, **c** IHC for ACTH, **d** Electron microscopy, original magnification ×  9700

**Fig. 6:**
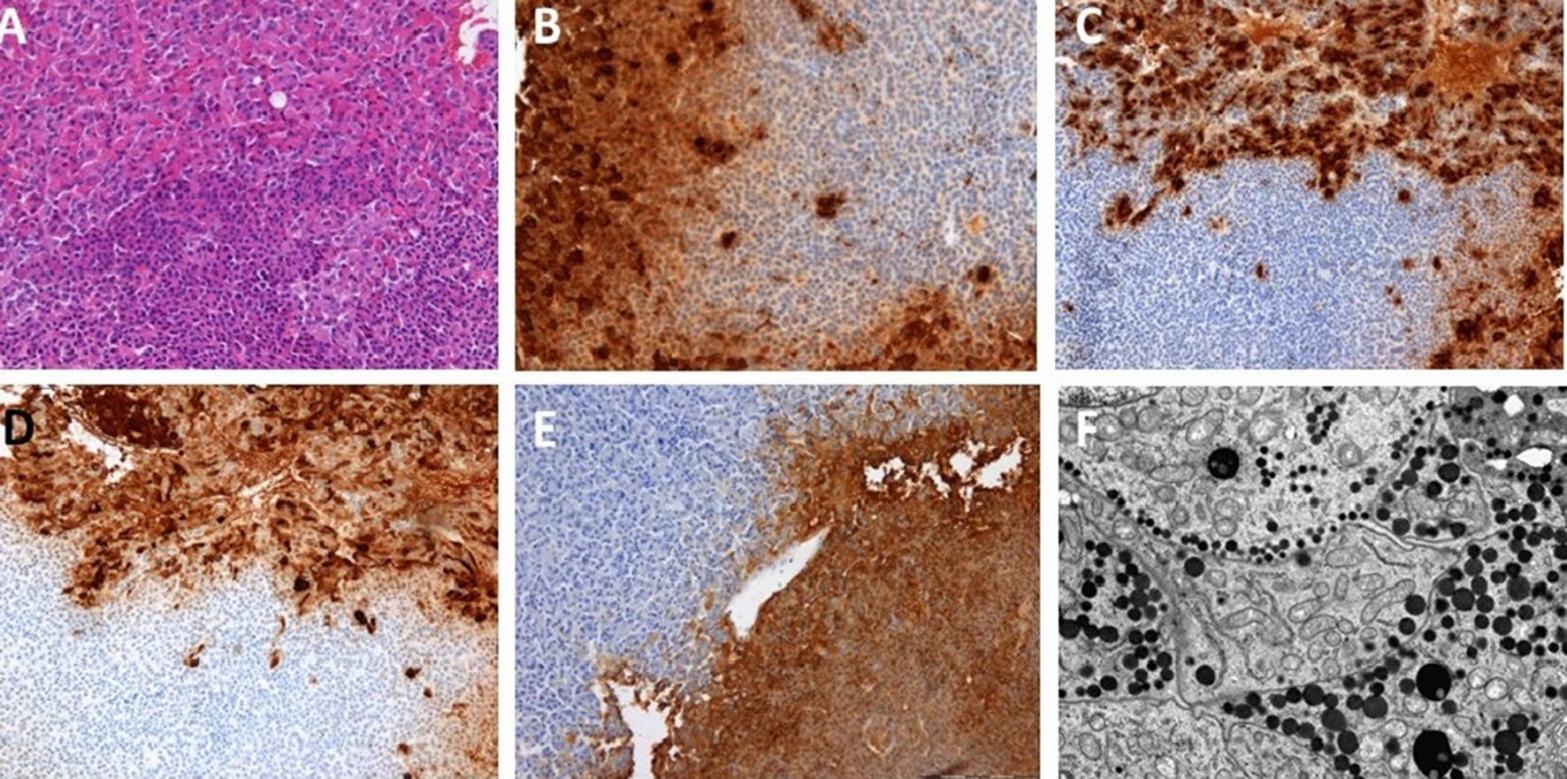
Contiguous double pituitary adenoma: case no. 17, acromegaly. Pathomorphological evaluation: **a** H&E, **b** IHC for GH, **c** IHC for PRL, **d** IHC for alpha subunit, **e** IHC for ACTH, **f** Electron microscopy original magnification × 9700

In the remaining patients the diagnosis of contiguous double pituitary adenoma was confirmed in the histopathological examination (Figs. 4,5 and 6).

**Fig. 7:**
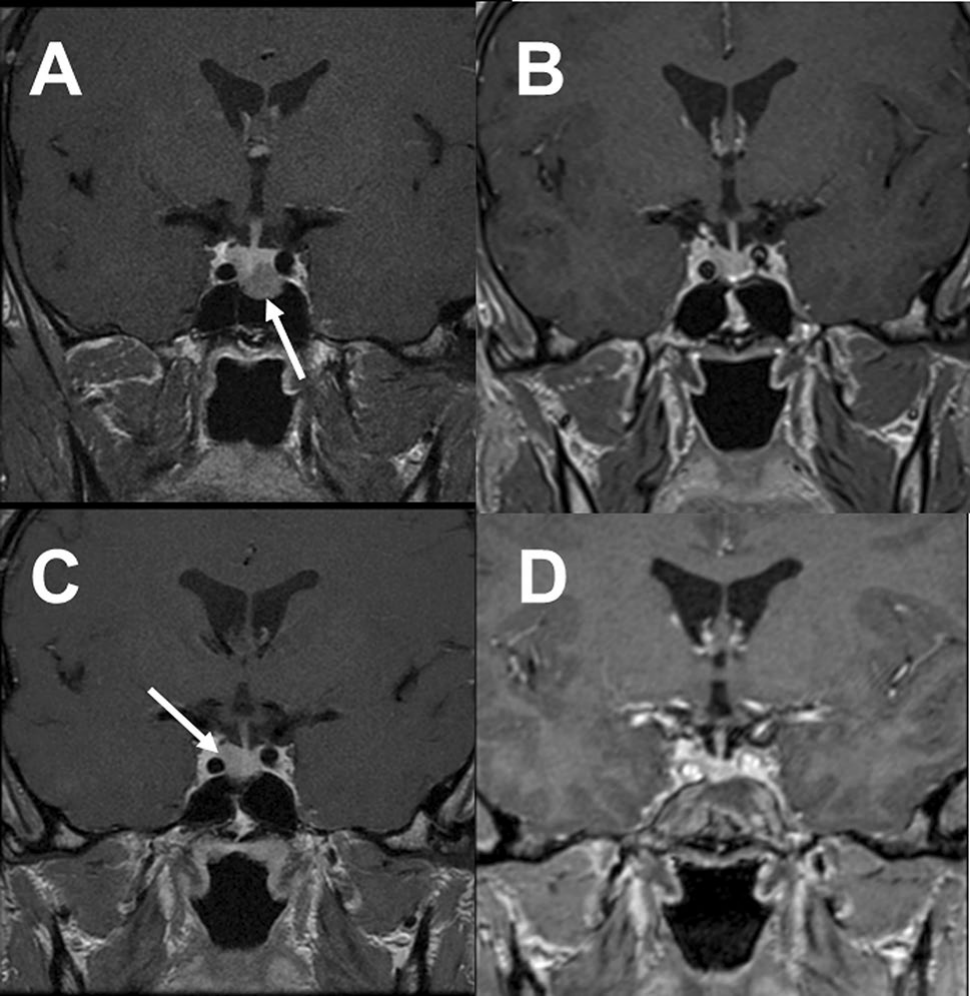
Case no. 12: **a** arrow indicates somatotroph pituitary macroadenoma removed with its pseudocapsule during first transsphenoidal operation (year 2008), **b** postoperative MR image (after first operation), remission of the acromegaly was achieved, **c** arrow indicates a new pituitary adenoma in the right lateral wing of the pituitary gland (year 2011), **d** postoperative MR image (after second operation)

In one patient (case 12) a second, histopathologically distinct pituitary adenoma was operated on three years after excision of the first adenoma, as mentioned before (Fig. 7).

**Fig. 8:**
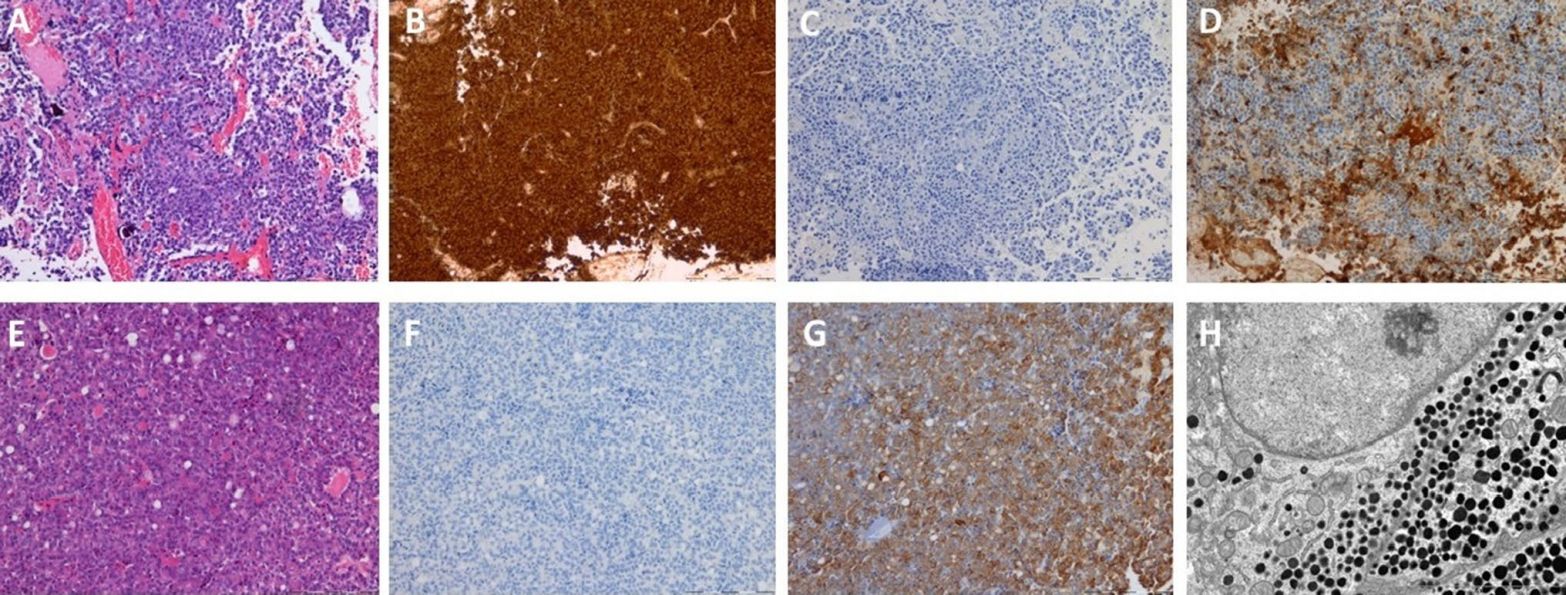
Separate tumors identified intraoperatively with different histopathological diagnosis: case no. 19, Cushing disease. Pathomorphological evaluation: I. Tumor on the right side: **a** H&E, **b** IHC for PRL, **c** IHC for ACTH, **d** IHC for alpha subunit; II. Tumor on the left side: **e** H&E, **f** IHC for PRL, **g** IHC for ACTH, **h** Electron microscopy, original magnification × 9700

One patient (case 19) was admitted to our neurosurgical unit with endocrinologically established diagnosis of ACTH dependent hypercortisolemia. The preoperative 3 T MR imaging with SPGR sequences revealed no visible abnormalities. To further establish the diagnosis of hypercortisolemia the patient underwent bilateral inferior petrosal sinus sampling and the pituitary source of ACTH was confirmed. The ratio of ACTH concentrations in both inferior petrosal sinuses was suggestive for a right-sided pituitary lesion. The patient was operated on through a transsphenoidal approach. Opening of the sellar floor at the right side and sellar exploration were performed. Intraoperatively, a mass consistent with microadenoma was encountered and excised. However, early morning postoperative cortisol levels indicated lack of Cushing’s disease remission. Therefore two days after the first procedure the patient was operated on again and sellar exploration on the left side was performed. A mass, 3 mm in size, was found and resected with its pseudocapsule. This time, the nadir morning serum cortisol level was below 1.8 µg/dL. The immediate remission of Cushing’s disease was achieved and hydrocortisone supplementation was administered. The histopathological examination revealed two distinct pituitary adenomas: first specimen stained positively for PRL, while the second specimen for ACTH (Fig. 8). The details of histopathological findings of the excised tumors are presented in Table 4.

**Table 4:** Histopathological evaluation of excised double pituitary adenomas

Case no	Clinical symptoms	IHC staining											Ultrastructural diagnosis (EM)
		GH	PRL	ACTH	TSH	FSH	LH	α-sub	p53 (%)	sstr2A	sstr5	Ki-67 (%)	
Separate tumors identified in the MRI and intraoperatively with different histopathological diagnosis													
1	Acromegaly	+	±	−	−	−	−	+	−	C+	C++	−	Densely granulated somatotroph adenoma
		−	±	−	−	±	+	−	−	−	C+	~ 3	Gonadotroph adenoma
2	Acromegaly	+	±	−	−	−	−	−	< 1	C+	C+	< 1	Sparsely granulated somatotroph adenoma
		+	±	−	−	−	−	−	−	C±	C+	< 1	Densely granulated somatotroph adenoma
3	Acromegaly	+	−	−	−	−	−	−	< 3	M++	C++	< 1	Densely granulated somatotroph adenoma
		−	+	−	−	−	−	−	< 1	C+	C++	< 1	Sparsely granulated lactotroph adenoma
4	Acromegaly, subclinical hypercortiso−lism	+	±	−	−	−	−	+	−	M++	C++	< 1	Densely granulated somatotroph adenoma
		−	−	+	−	−	+	+	N/A	C+	C++	< 1	Sparsely granulated corticotroph adenoma
5	Acromegaly	+	±	−	−	−	−	−	< 1	M++ C+	C±	< 1	Densely granulated somatotroph adenoma
		−	−	−	−	+	+	−	−	M+ C+	C±	< 1	Gonadotroph adenoma
8	NFPA	−	−	+	−	−	−	−	−	N/A	N/A	< 1	Silent sparsely granulated corticotroph adenoma
		−	−	+	−	±	−	±	−	N/A	N/A	< 1	
9	Acromegaly	−	−	−	−	−	+	−	< 1	−	−	< 3	Gonadotroph adenoma
		+	±	−	−	−	±	+	~ 3	M++	C±	< 1	Densely granulated somatotroph adenoma
19	Cushing disease	−	+	−	−	−	−	−	N/A	N/A	N/A	< 1	Prolactinoma (EM not assessed)
		−	−	+	−	−	−	−	N/A	N/A	N/A	< 1	Densely granulated corticotroph adenoma
18	Acromegaly	+	−	−	−	−	−	++	< 1	M+ C+	C±	< 1	Densely granulated somatotroph adenoma
		−	−	−	−	±	+	±	< 1	C±	−	~ 3	Gonadotroph adenoma
Two tumors with different histopathological findings in the same patient in three years’ time interval													
12	Acromegaly	+	−	−	−	−	−	−	< 1	M++ C+	C±	< 1	Densely granulated somatotroph adenoma
	NFPA	−	−	−	−	+	+	+	< 1	C+	C±	< 3	Gonadotroph adenoma
Contiguous double pituitary adenomas													
6	Cushing disease	−	+	−	−	−	−	−	< 1	N/A	N/A	< 3	Sparsely granulated lactotroph adenoma
		−	−	+	−	−	−	−	< 1	N/A	N/A	< 3	
7	Cushing disease	+	−	−	−	−	−	−	> 3	N/A	N/A	< 3	Densely granulated corticotroph adenoma
		−	−	+	−	−	−	−	> 3	N/A	N/A	< 3	
10	Acromegaly	+	−	−	−	−	−	−	−	C±	−	< 1	Mammosomatotroph adenoma
		+	±	−	−	−	−	+	< 1	C±	−	< 1	
11	Acromegaly	+	−	−	−	−	−	−	< 1	C+ M+	C±	< 1	Densely granulated somatotroph adenoma
		−	−	−	−	+	−	+	~ 2	−	−	< 1	
14	Secondary hyperthyroi−dism	+	−	−	++	−	−	++	−	M++	C+	< 1	Pit−1 positive plurihormonal adenoma
		−	−	−	−	+	+	−	−	C±	−	< 1	
15	Acromegaly	+	−	−	−	−	−	−	−	C+	−	< 1	Mixed somatotroph−lactotroph cell adenoma
		−	++	−	−	−	−	±	−	−	−	< 3	
17	Acromegaly	++	+	−	−	−	−	++	< 1	C+	−	< 1	Mixed somatotroph−lactotroph cell adenoma
		−	−	+	−	−	−	−	< 3	−	−	< 1	
20	NFPA	−	+	−	+	−	−	+	N/A	N/A	N/A	< 1	Pit−1−positive plurihormonal adenoma
		−	−	−	−	−	−	−	N/A	N/A	N/A	> 5	
Separate tumors identified in the MRI and intraoperatively with similar histopathological diagnosis													
16	NFPA	−	−	−	−	+	+	+	N/A	N/A	N/A	< 1	Gonadotroph adenoma
		−	−	−	−	+	+	+	N/A	N/A	N/A	< 1	Gonadotroph adenoma
13	NFPA	−	−	−	−	+	+	+	N/A	N/A	N/A	< 1	Oncocytic gonadotroph adenoma
		−	−	−	−	+	+	+	N/A	N/A	N/A	< 1	Oncocytic gonadotroph adenoma
21	Acromegaly, Carney syndrome	+	±	−	−	−	−	−	N/A	N/A	N/A	< 3	Densely granulated somatotroph adenoma
		+	±	−	−	−	−	−	N/A	N/A	N/A	< 3	Densely granulated somatotroph adenoma
22	NFPA	−	−	−	−	−	−	±	N/A	N/A	N/A	< 3	Gonadotroph adenoma
		−	−	−	−	−	+	+	N/A	N/A	N/A	< 3	Gonadotroph adenoma

*GH* growth hormone, *PRL* prolactin, *NFPA* nonfunctioning adenoma, *sstr* somatostatin receptor subtypes (2A and 5), *M* membrane reaction, *C* cytoplasmic reaction, *N/A* not assessed, *EM* electron microscopy

**Fig. 9:**
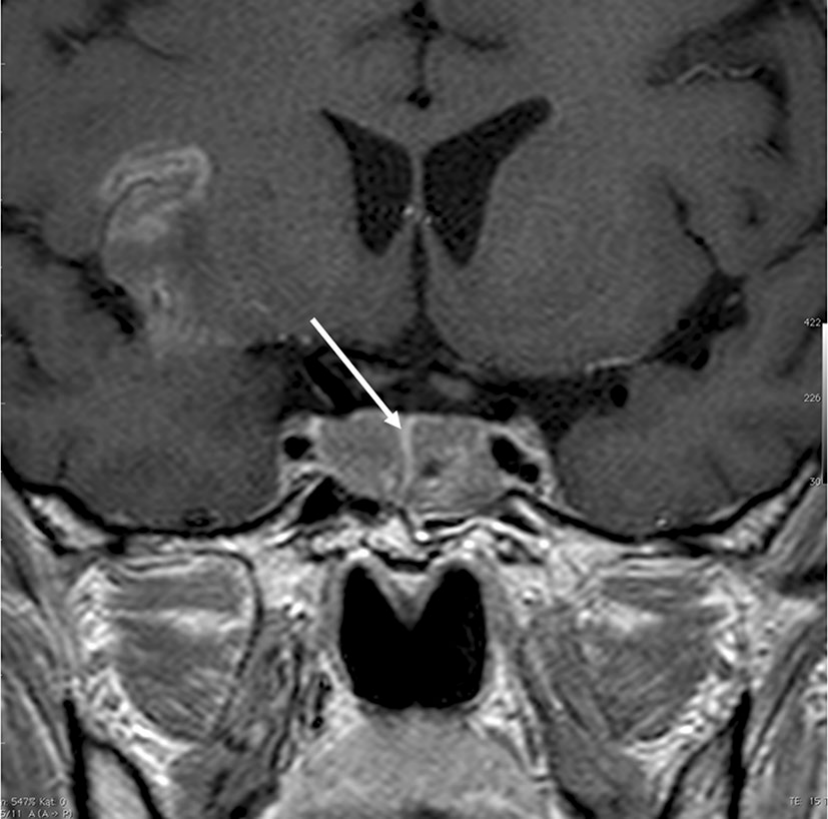
Case no. 13, T1 weighted image after GD-DTPA. Separate tumors were identified in the MR imaging and intraoperatively, the arrow indicated flattened pituitary gland

**Fig. 10:**
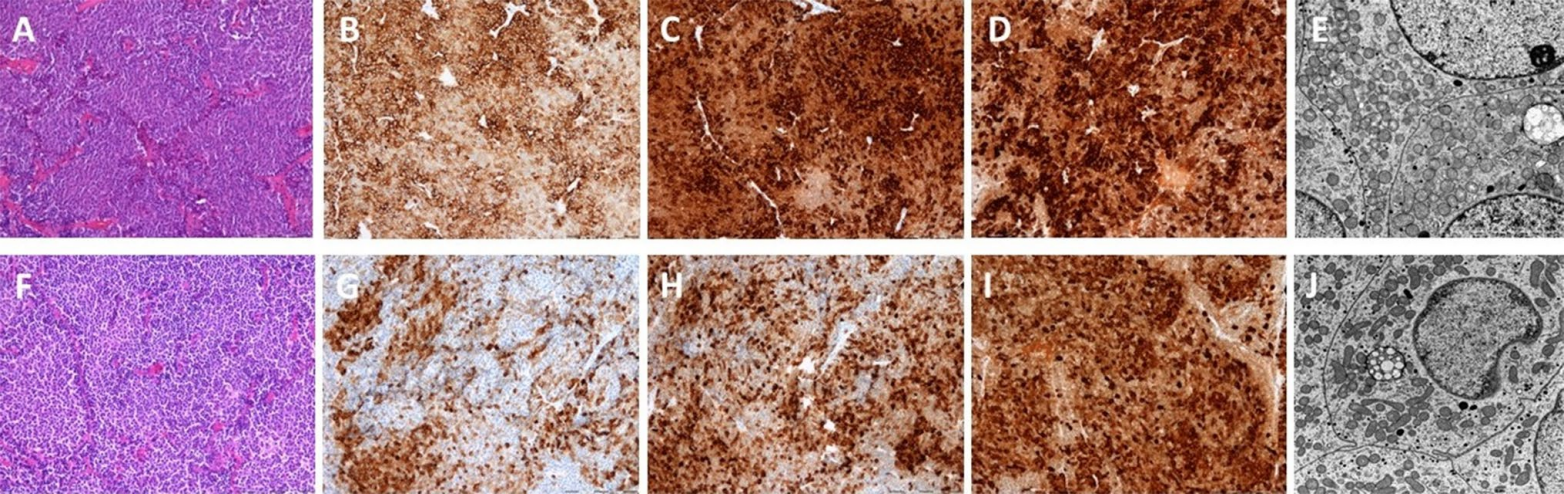
Separate tumors identified in the MRI and intraoperatively with similar histopathological diagnosis: case no. 13, NFPA. Pathomorphological evaluation: I. Tumor on the right side: **a** H&E, **b** IHC for FSH, **c** IHC for LH, **d** IHC for alpha subunit, **e** Electron microscopy, original magnification × 9700; II. Tumor on the left side: **f** H&E, **g** IHC for FSH, **h**IHC for LH, **i** IHC for alpha subunit, **j** Electron microscopy, original magnification × 9700

In two cases (13 and 16) the preoperative MRI imaging and intraoperative findings revealed clear separation of two tumors (Fig. 9). However, they presented similar histopathological findings (Table 4; Fig. 10). None of these adenomas presented a horseshoe pattern on the preoperative MRI or intraoperatively, which may have led to a false diagnosis of double pituitary adenoma [10].

**Fig. 11:**
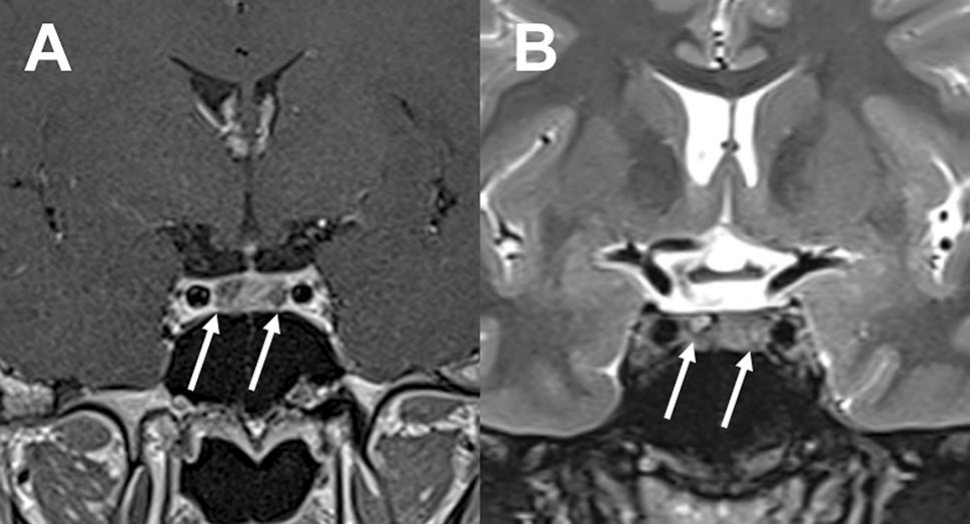
Case no. 21, **a** and **b** coronal T1 weighted after GD-DTPA (left image) and T2 weighted (right image) MR imaging of the pituitary gland of the patient with diagnosed Carney complex. Arrows indicate two clearly separate microadenomas confirmed during operation and histological examination

**Fig. 12:**
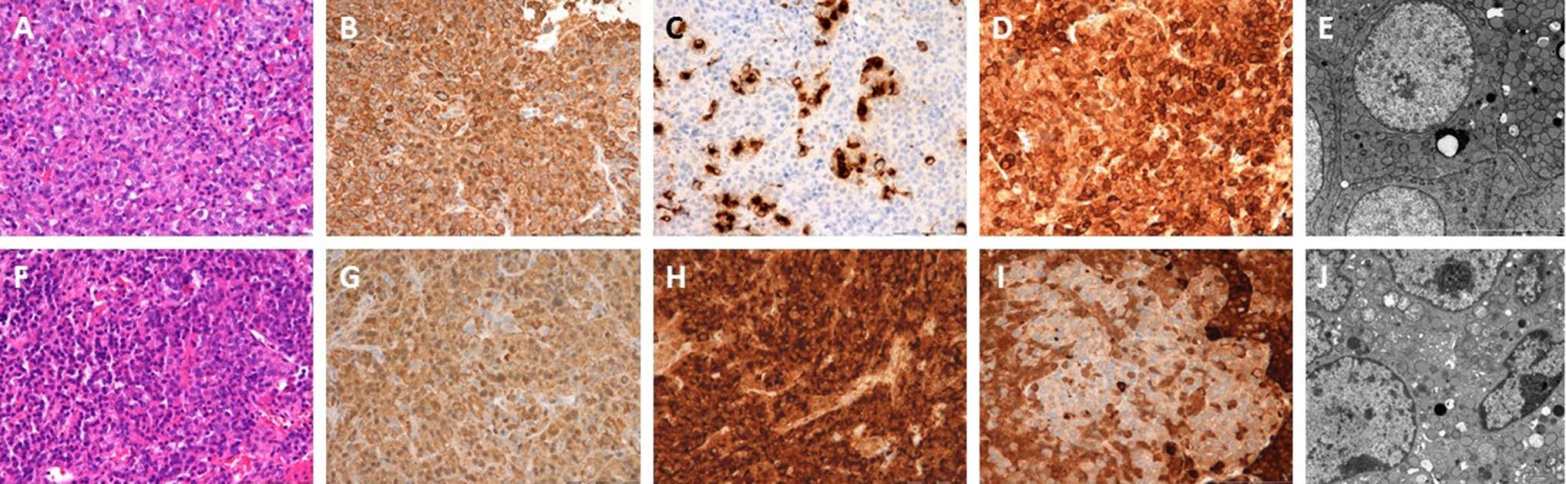
Separate tumors identified in the MRI and intraoperatively with similar histopathological diagnosis: case. no 21, acromegaly, Carney complex. Pathomorphological evaluation: I. Tumor on the right side: **a** H&E, **b** IHC for GH, **c** IHC for PRL, **d** IHC for alpha subunit, **e** Electron microscopy, original magnification × 7400; II. Tumor on the left side: **f** H&E, **g** IHC for GH, **h** IHC for PRL, **i** IHC for alpha subunit, **j** Electron microscopy, original magnification × 9700

One of the patients with multiple pituitary adenomas (case no. 21) presented with the symptoms of Carney complex. The diagnosis was confirmed by clinical signs and symptoms as well as genetic test. Prior to pituitary surgery he was operated on because of Cushing’s syndrome caused by right adrenal adenoma and cardiac myxoma in the right atrium. His pituitary 3 T MR imaging suggested double clearly separated pituitary adenomas and they were confirmed during surgery and pathological examination (Figs. 11 and 12; Table 4).

All the patients were followed-up with control MR imaging and early and late hormonal status was assessed. First post-operative MR imaging was routinely performed 3 months after the procedure. The endocrinological assessment is presented in the Supplementary Table 1. The average time of follow-up was 74.2 months. The remission of anterior pituitary hormone syndrome was achieved in all patients except two cases of acromegaly (cases no. 2 and 11). There were no late recurrences in the follow-up period.

## Discussion

Double and multiple pituitary adenomas are reported in 0.9% of random pituitary autopsy samples [10]. Their prevalence in surgical case series ranges from 0.2 up to 2.6% of resected pituitary adenomas [11, 12]. The increasing recognition of multiple pituitary adenomas is likely attributable to the expanded application of high field MR scanners for pituitary lesions imaging and modern immunocytological methods for the diagnosis of these tumors [13]. The pathogenesis of double and multiple pituitary adenomas is explained by multiple-hit or transdifferentiation theories [5]. The first theory assumes coincidental monoclonal expansion of two distinct genetically mutated pituitary cell types and is supported by distinct tumor capsules at microdissection of surgical specimens [6]. The transdifferentiation theory is based on the ability of the cells of one pituitary adenoma to transdifferentiate into another cell type. This theory is supported by genetic profiling and transcription factor expression in double pituitary adenomas [5, 14].

In our case series, the diagnosis of multiple pituitary adenoma was established in 22 cases (11 male and 11 female) out of the total of 3270 resected pituitary adenomas (0.67%). Thus the reported incidence is similar to the above mentioned data in literature, however we did not observe the predominance for female patients as was reported in a recent review by Ogando-Rivas et al*.* [15]. There was one case of an asynchronous separate and distinct double pituitary adenoma in our case series. A similar case of an asynchronous double pituitary adenoma with different morphological and ultrastructural features was presented by Thodou et al*. *[8]. Of the synchronous multiple pituitary adenomas, 11 were clearly separate in the preoperative neuroradiological imaging and intraoperatively, 9 were contiguous tumors resected as one mass and in one case the preoperative diagnostic imaging was negative, however operative exploration reveled two distinct microadenomas.

The most of the reported multiple pituitary adenomas in the literature were found in the setting of acromegaly as in a series presented by Sano et al. [16] where all of the six cases of double pituitary adenomas were positive for GH. In a review of multiple pituitary adenomas by Ogando-Rivas et al*.*, the most commonly reported plurihormonal adenomas jointly expressed were the GH–PRL–TSH+FSH–LH, GH–PRL–TSH+ACTH, and ACTH+FSH–LH groups [15]. The results presented in this work match this tendency. The majority of presented lesions were hormonally active. The most frequent clinical syndrome was acromegaly (12/22) followed by Cushing disease (3/22) which is consistent with the previously published data [15, 17]. The most frequent setting was the coexistence of GH-secreting adenoma with non-functioning gonadotroph adenomas, which occurred in 5 cases.

In Cushing’s disease patients, ACTH-secreting adenomas coexisted with PRL-secreting tumors in two cases and with GH-secreting adenoma in one case. In a study by Ratliff et al. [6] double pituitary adenomas were identified in 13 out 660 patients operated on with the diagnosis of Cushing disease. Prolactinomas were the most common incidental tumors in this study which is in accordance with our findings. Additionally, synchronous ACTH-secreting pituitary adenomas might occur in anterior lobe or in pituitary stalk further hampering the treatment [6]. Taking this into account, recognition and exact localization of double pituitary adenomas prior to surgery are essential to avoid the failure of surgery by missing the causative lesion which is of special importance in the course of treating Cushing’s disease caused by microadenomas [5]. Such a scenario happened in the case no. 19 where multiple pituitary adenomas were not suspected prior to surgery. This led to the lack of immediate remission of Cushing’s disease after the first procedure. It was confirmed by the early postoperative hormonal evaluation and pathological assessment. Removed microadenoma was classified as a PRL-secreting tumor. Interestingly though, the results of BIPSS guided the surgical exploration in the false direction, further blurring the clinical presentation. The challenges of detecting small synchronous multiple pituitary adenomas and resulting failure of treatment have been previously reported as well as the false negative results of adenoma lateralization in BIPSS procedure [6, 18–20]. However, as mentioned before, one should remember that PRL-secreting microadenomas are the most common incidental lesions coexisting with corticotroph pituitary tumors [6].

MRI imaging has an unquestionable importance in detecting multiple separated pituitary adenomas. It is widely known that the meticulous preoperative localization of a pituitary microadenoma is associated with greater surgical efficacy, especially in Cushing’s disease [21]. It might be difficult because of the petite of some tumors [22]. Modern, high field MRI scanners offer greater sensitivity in the pituitary tumors detection. Furthermore, special protocols of pituitary lesions imaging such as a dynamic and/or SPGR techniques improves detection of minute pituitary adenoma but their specificity are low [23]. In a particular situations, BIPSS is a method of choice in the diagnosis of pituitary dependent hypercortisolemia and detection and localization of corticotroph pituitary tumors [21]. Metionine-PET/MR and intraoperative ultrasound of the pituitary gland might offer additional information in localizing challenging cases [24, 25]. Detailed and full preoperative endocrine assessment is also crucial as it may suggest co-occurrence of multiple adenomas.

In our series we presented a case of Carney complex. Two clearly separated pituitary microadenomas were suspected in the preoperative imaging and were confirmed intraoperatively. The histopathological examination revealed densely granulated somatotroph adenoma in both of the excised specimen. This is consistent with a case series of seven Carney complex patients with GH-secreting pituitary adenomas presented by Lonser et al.[26]. Three out of them had multiple tumors visible on preoperative MR imaging and identified during surgery. Their pathological examination confirmed multiple pituitary adenomas with concurrent mammosomatotroph hyperplasia. We did not observed any hyperplasia in the removed tissue in our case.

Identification of clearly separated multiple pituitary macroadenomas on MRI imaging seems to be easy because of their different signal intensity on T1 and T2 weighted MR imaging as was detected in 11 patients in our study group. Part of the difficulty is due to the failure of diagnostic imaging of the microadenomas. MR imaging can detect microadenomas of 2–3 mm with a sensitivity of only 85% respectively [23]. In our material the 3 T MR imaging with dynamic and SPGR sequences failed to detect double microadenoma in case no. 19.

Recognition of contiguous double adenomas is more difficult and it is based on the correct interpretation of the immunohistochemical localization of disparate pituitary hormones in different adenomatous cells regardless of endocrinological and neuroradiological assessment. In our study group the diagnosis of contiguous double pituitary adenomas was confirmed in nine cases.

In some cases, electron microscopy may play a pivotal role to accurately determine the cell type comprising a tumor and to set down the diagnosis of multiple pituitary adenomas [14]. Recent years have seen an increasing application of the pituitary transcription factors (Pit-1, Tpit and SF-1) to accurately determine the adenoma cells type and distinguish separate primary lesions that are collision tumors or divergent differentiation of a single lesion [14, 17]. Both methods are very helpful in differentiation of multiple monoclonal adenomas from single plurihormonal adenomas.

## Conclusions

Double pituitary adenomas are a very rare entity. However, in clinical setting, they can pose a significant challenge. Careful inspection of preoperative MR imaging and detailed hormonal assessment are essential for correct diagnosis and successful surgical treatment, especially in synchronous separated adenomas. Special attention should be paid in patients diagnosed for Cushing’s disease when neuroradiological imaging reveals no visible lesions. Thorough pathomorphological examination according to WHO recommendation is essential for establishing correct diagnosis of contiguous double pituitary adenomas. Ultrastructural analysis regardless of immunocytodifferentiation may be pivotal to confirm an exact diagnosis in such cases.

## Electronic supplementary material

Below is the link to the electronic supplementary material.

Supplementary file1 (XLSX 13 kb)
